# Supplementary material for: Dysregulation of Metabolism and Proteostasis in Skeletal Muscle of a Presymptomatic Pompe Mouse Model
Source: Cells. 2023 Jun 11;12(12):1602. doi: 10.3390/cells12121602 (PMC10297364; doi:10.3390/cells12121602)
Supplement: Supplementary file 1 [file cells-12-01602-s001.zip › cells-2425255-supplementary.pdf]

cells

Supplementary Materials

Table S1: Overview of dysregulated proteins found in six-weeks-old Pompe mice quadriceps muscle

UniProt-AC	FC	P-Val	UniProt-AC	FC	P-Val	UniProt-AC	FC	P-Val
P16858	1.08	0.01	P35979	1.32	0.01	Q9Z1E4	1.38	0.00
P09411	1.09	0.01	P11404	1.32	0.00	P62192	1.38	0.03
P28650	1.15	0.03	Q3THW5	1.32	0.01	Q9Z2U0	1.39	0.00
P52480	1.18	0.05	Q9D6R2	1.32	0.02	P08030	1.39	0.02
Q01768	1.19	0.02	P53810	1.33	0.02	Q9D855	1.39	0.01
Q64521	1.20	0.04	P62806	1.33	0.01	Q8K3J1	1.39	0.02
P62911	1.20	0.04	P62259	1.33	0.01	P60867	1.39	0.02
P15864	1.21	0.02	P97807	1.33	0.01	P48774	1.39	0.01
Q6ZQ73	1.22	0.01	Q62000	1.33	0.04	Q9CRB6	1.39	0.05
P56375	1.22	0.01	P56135	1.33	0.04	Q91VM9	1.39	0.04
Q3MI48	1.22	0.01	P45377	1.33	0.02	Q9DCJ5	1.39	0.00
Q9QZQ8	1.23	0.01	Q922J3	1.34	0.01	P62281	1.39	0.03
P32848	1.24	0.01	P63323	1.34	0.01	P62852	1.39	0.01
P80318	1.24	0.05	P63017	1.34	0.02	O09061	1.39	0.01
Q9D2G2	1.24	0.02	Q9CR68	1.34	0.02	Q9DCS9	1.40	0.01
Q9CQ69	1.24	0.04	Q99JY0	1.34	0.02	Q9CQZ5	1.40	0.00
Q6P1F6	1.25	0.02	Q91VD9	1.34	0.00	Q8K1Z0	1.40	0.04
P52503	1.25	0.03	Q8QZT1	1.34	0.01	A6X935	1.40	0.03
P68037	1.25	0.01	P14733	1.35	0.00	Q60675	1.40	0.02
Q9CZU6	1.25	0.01	P51881	1.35	0.00	Q3U1J4	1.40	0.00
P70670	1.26	0.02	Q8R3G9	1.35	0.02	Q06185	1.40	0.01
Q9WTP6	1.26	0.05	Q9Z1N5	1.35	0.00	Q9CPQ8	1.40	0.00
Q9WUM5	1.26	0.00	Q6ZWV7	1.36	0.03	P02463	1.40	0.02
Q9D0M5	1.26	0.04	P35486	1.36	0.00	P48678	1.40	0.01
Q76MZ3	1.27	0.03	O88456	1.36	0.02	P45591	1.41	0.01
Q9D8N0	1.27	0.02	P55264	1.36	0.04	Q9DB20	1.41	0.00
Q60931	1.28	0.04	P19253	1.36	0.00	P67778	1.41	0.01
O55126	1.28	0.00	Q9CXV1	1.36	0.03	P12815	1.41	0.03
Q99KI0	1.29	0.03	Q9JJV2	1.36	0.02	Q9D0M3	1.41	0.00
Q9D3D9	1.29	0.01	Q9CQ62	1.37	0.03	P25444	1.41	0.02
P63328	1.29	0.04	Q6ZWY3	1.37	0.05	Q99020	1.41	0.02
O08749	1.29	0.00	Q9DCX2	1.37	0.01	P62827	1.41	0.01
P08249	1.29	0.03	Q7TMF3	1.37	0.01	P50544	1.41	0.02
P68040	1.29	0.03	Q924X2	1.38	0.02	P10493	1.42	0.00
P63001	1.30	0.05	Q61584	1.38	0.03	O88712	1.42	0.03
P47963	1.30	0.05	P48962	1.38	0.04	O35350	1.42	0.00
Q9CPQ1	1.31	0.04	P19783	1.38	0.02	P62897	1.42	0.01
Q9CQC7	1.31	0.02	P97351	1.38	0.01	Q03265	1.42	0.00
Q6NT99	1.32	0.04	Q70IV5	1.38	0.01	P35980	1.42	0.02

UniProt-AC	FC	P-Val	UniProt-AC	FC	P-Val	UniProt-AC	FC	P-Val
Q9Z2I9	1.42	0.01	P47962	1.46	0.03	Q9CQQ7	1.52	0.03
Q3U962	1.42	0.05	O55234	1.46	0.00	Q922B2	1.52	0.02
Q8BG32	1.42	0.04	Q6ZWV3	1.47	0.01	P05201	1.52	0.02
P14131	1.42	0.00	Q9CQR4	1.47	0.02	Q99LY9	1.52	0.01
O54724	1.42	0.01	Q9D8B4	1.47	0.03	Q61171	1.52	0.00
Q61425	1.42	0.00	Q9Z1P6	1.47	0.02	P62900	1.52	0.01
P62702	1.42	0.02	P11499	1.47	0.04	P62082	1.53	0.01
P61358	1.43	0.01	O08709	1.47	0.00	O70251	1.53	0.04
Q9DB77	1.43	0.00	Q8R404	1.47	0.00	P22599	1.53	0.03
Q9CQ75	1.43	0.01	P00405	1.47	0.00	Q9D967	1.53	0.03
P48758	1.43	0.02	O70435	1.47	0.00	Q8VEK3	1.53	0.02
Q8R127	1.43	0.05	A2AUC9	1.47	0.03	Q9JI91	1.53	0.02
Q8BMF4	1.43	0.00	Q9D051	1.47	0.00	Q9CYT6	1.53	0.00
Q8BH59	1.43	0.00	Q6ZWX6	1.47	0.01	Q60597	1.53	0.00
Q9D0I9	1.43	0.00	P17742	1.48	0.01	P35282	1.54	0.04
Q8R1I1	1.43	0.05	P62889	1.49	0.01	Q04857	1.54	0.05
Q9CQA3	1.43	0.00	Q9D8E6	1.49	0.01	P09671	1.54	0.00
P53026	1.43	0.01	Q9D2M8	1.49	0.02	P02469	1.54	0.00
P62245	1.44	0.00	Q8VEM8	1.49	0.01	Q3TL44	1.54	0.01
P20029	1.44	0.00	Q99MQ4	1.49	0.02	P62754	1.54	0.04
P62717	1.44	0.01	Q91YT0	1.49	0.00	P67984	1.54	0.01
P56391	1.44	0.00	Q8CGY6	1.49	0.00	Q9D7X3	1.54	0.00
Q99L13	1.44	0.01	P31001	1.49	0.01	Q8BGH2	1.55	0.02
Q11011	1.44	0.04	P61979	1.49	0.02	P51410	1.55	0.00
Q9ERS2	1.44	0.01	Q61292	1.50	0.01	P62242	1.56	0.00
P63038	1.44	0.01	Q60930	1.50	0.00	Q7TMK9	1.56	0.02
Q91WS0	1.44	0.01	Q9CR61	1.50	0.03	P05202	1.56	0.01
P53994	1.44	0.03	P56480	1.50	0.00	P62830	1.57	0.02
Q9WU78	1.44	0.00	P70195	1.50	0.01	Q9CQJ8	1.57	0.00
P14115	1.45	0.04	P13020	1.50	0.02	Q64310	1.57	0.00
Q9Z2U1	1.45	0.01	Q99JB8	1.50	0.00	Q78IK2	1.57	0.01
Q9CZ13	1.45	0.00	Q9R1P4	1.51	0.01	Q93092	1.58	0.04
P61255	1.45	0.02	Q8CAQ8	1.51	0.00	Q9D1M0	1.58	0.04
Q6ZWN5	1.45	0.00	Q8CGK3	1.51	0.01	P42932	1.58	0.02
Q60668	1.45	0.03	Q9CZX8	1.51	0.01	Q9D0R2	1.58	0.02
P70333	1.45	0.02	P62908	1.51	0.00	P51885	1.58	0.01
P07724	1.46	0.04	Q01853	1.51	0.00	P54071	1.58	0.01
P99029	1.46	0.00	Q64105	1.51	0.02	Q9CWJ9	1.58	0.01
P62751	1.46	0.04	P14148	1.51	0.00	Q68FD5	1.59	0.00
P08122	1.46	0.02	Q91WD5	1.51	0.02	O08756	1.60	0.03
Q9ES74	1.46	0.01	P46471	1.52	0.00	P14824	1.60	0.00
P26443	1.46	0.00	Q8K2B3	1.52	0.00	P11531	1.61	0.00
O08539	1.46	0.00	Q9DC69	1.52	0.00	P45952	1.61	0.00

UniProt-AC	FC	P-Val
O35206	1.61	0.00
P63085	1.61	0.03
Q9CPR4	1.61	0.00
Q9DCZ1	1.61	0.01
Q61554	1.61	0.02
P46638	1.61	0.00
P21812	1.62	0.02
Q61234	1.62	0.02
Q9CQE8	1.62	0.04
O35129	1.62	0.00
P60335	1.62	0.00
P14869	1.62	0.00
Q99MR9	1.63	0.04
Q9CR62	1.63	0.02
P84104	1.63	0.02
P62880	1.63	0.02
Q9CR09	1.63	0.05
Q9CXS4	1.64	0.01
P07356	1.64	0.00
P47911	1.64	0.01
O35864	1.64	0.03
Q9JK92	1.64	0.00
Q9D6U8	1.64	0.01
Q8BFR5	1.64	0.00
P38647	1.64	0.00
Q9WVJ2	1.65	0.01
Q05793	1.65	0.03
Q08857	1.65	0.02
P56695	1.65	0.02
Q8BKC5	1.65	0.02
P03921	1.65	0.04
Q66JS6	1.65	0.05
Q70KF4	1.65	0.02
Q9Z2Z6	1.66	0.02
Q99LC3	1.66	0.01
Q99KB8	1.66	0.02
P47934	1.66	0.00
P62983	1.66	0.01
Q3UIU2	1.66	0.02
Q3TC72	1.66	0.02
Q9CZB0	1.66	0.02
P62301	1.67	0.03
Q9R0Q7	1.67	0.02

UniProt-AC	FC	P-Val
Q921G7	1.67	0.04
Q9CRB9	1.67	0.01
Q3TEA8	1.67	0.01
O08528	1.68	0.00
Q61598	1.68	0.01
Q9CQH3	1.68	0.01
Q9D8W5	1.68	0.02
P62855	1.69	0.02
P07901	1.69	0.01
P09405	1.70	0.01
Q9CPP6	1.70	0.00
Q9QZZ6	1.70	0.01
Q791V5	1.71	0.00
Q99MN1	1.71	0.05
P01027	1.71	0.03
Q9JM76	1.71	0.04
P16546	1.71	0.02
Q8BFZ3	1.71	0.01
P97384	1.72	0.01
O08638	1.72	0.03
P09528	1.72	0.01
P21981	1.73	0.04
P14685	1.73	0.00
Q99L47	1.73	0.01
O55142	1.73	0.03
Q9QWL7	1.73	0.01
P97355	1.74	0.01
P48036	1.74	0.03
Q6IRU5	1.74	0.01
O55222	1.74	0.03
Q8BWT1	1.75	0.01
P16045	1.75	0.02
P82347	1.75	0.02
Q9D0K2	1.75	0.01
P62196	1.75	0.03
Q8CDN6	1.75	0.02
P15089	1.76	0.02
P42669	1.76	0.05
O70325	1.76	0.05
P41216	1.76	0.00
Q8C7E7	1.77	0.02
P42125	1.77	0.02
P16015	1.77	0.00

UniProt-AC	FC	P-Val
Q9D1G1	1.78	0.04
Q8BZA9	1.78	0.00
P62334	1.79	0.01
P68369	1.79	0.00
P00158	1.79	0.02
P68368	1.79	0.00
P32020	1.79	0.03
P23953	1.79	0.04
Q9DB73	1.80	0.03
Q99PT1	1.80	0.02
Q922F4	1.80	0.04
Q99MN9	1.81	0.03
Q9DB60	1.81	0.01
Q8BG05	1.81	0.00
P63101	1.82	0.00
P27546	1.82	0.04
P82348	1.82	0.03
P09055	1.82	0.00
O35344	1.84	0.03
P61161	1.84	0.03
Q9CZM2	1.85	0.01
P99026	1.85	0.01
Q8K4Z3	1.86	0.00
Q9DBP5	1.86	0.02
Q9DCW4	1.86	0.03
O08677	1.86	0.03
Q8VDM4	1.87	0.00
Q61699	1.87	0.02
Q99JI4	1.87	0.00
Q6P8J7	1.87	0.01
P54775	1.88	0.04
A2AMM0	1.88	0.01
Q9R0P5	1.89	0.00
P10639	1.89	0.02
O35459	1.90	0.01
P51174	1.90	0.00
O35658	1.90	0.01
P47955	1.90	0.03
Q6PER3	1.91	0.02
O08553	1.91	0.01
Q91WK5	1.91	0.03
Q00896	1.92	0.02
O88545	1.94	0.01

UniProt-AC	FC	P-Val
Q9JMA1	1.94	0.01
P60710	1.94	0.01
Q922R8	1.95	0.03
Q9CPU4	1.95	0.03
P51150	1.95	0.00
P08113	1.95	0.02
Q9CYR0	1.96	0.03
Q5SX40	1.96	0.02
Q9R1P1	1.97	0.02
P63073	1.97	0.00
P02535	1.99	0.02
P22315	1.99	0.03
P21614	1.99	0.01
Q9JI75	2.00	0.01
P50580	2.00	0.01
Q9WVH9	2.00	0.02
Q05816	2.01	0.01
Q9QWV4	2.02	0.03
Q62261	2.02	0.02
Q5DTJ9	2.02	0.01
Q9DB27	2.03	0.03
P59999	2.04	0.00
P26041	2.04	0.05
O70209	2.05	0.00
Q3THE2	2.05	0.01
Q9R062	2.06	0.00
Q8R4N0	2.06	0.02
Q60631	2.07	0.00
Q9DCD0	2.07	0.04
P62835	2.08	0.00
P35293	2.08	0.00
Q01339	2.08	0.01
E9PV24	2.09	0.03
P47811	2.10	0.02
Q91ZJ5	2.10	0.00
O08529	2.10	0.01
Q9DCS3	2.11	0.01
Q920M5	2.12	0.03
Q8BJY1	2.13	0.05
P10126	2.13	0.05
Q8C6K9	2.14	0.05
Q9R1P0	2.14	0.03
Q61166	2.14	0.03

UniProt-AC	FC	P-Val
O35855	2.14	0.02
P54923	2.14	0.03
P61961	2.15	0.04
Q7TNG5	2.15	0.03
P10605	2.15	0.01
Q62165	2.15	0.03
Q9JHU4	2.17	0.00
Q9D783	2.17	0.00
Q78IK4	2.17	0.02
P19157	2.19	0.01
P70398	2.20	0.02
P63094	2.20	0.00
Q6PHZ2	2.20	0.00
Q9QZ06	2.20	0.01
Q8BTM8	2.23	0.00
Q3ULJ0	2.24	0.01
Q8VHX6	2.25	0.00
P62849	2.25	0.00
Q922Q8	2.26	0.03
P27573	2.29	0.04
P57780	2.30	0.00
Q9CVB6	2.30	0.01
Q08642	2.30	0.04
P63321	2.31	0.03
Q8CIB5	2.32	0.02
Q8C0C7	2.32	0.00
Q5M8N4	2.33	0.02
Q78ZA7	2.34	0.03
P35762	2.34	0.01
Q9CY50	2.35	0.01
Q9JJZ2	2.35	0.00
P84091	2.37	0.04
Q99KQ4	2.38	0.00
Q9CQN1	2.38	0.00
Q8K1M6	2.40	0.00
Q9JMH6	2.40	0.01
P80316	2.40	0.04
P47753	2.41	0.01
Q8BWM0	2.41	0.03
P17427	2.43	0.03
P24472	2.43	0.01
Q9WVK4	2.43	0.05
P11352	2.45	0.03

UniProt-AC	FC	P-Val
P62960	2.45	0.02
Q9DB05	2.46	0.02
Q91V41	2.49	0.00
Q9WVA4	2.52	0.03
Q9ESL4	2.52	0.04
Q99JY9	2.54	0.00
P60843	2.55	0.00
Q91W90	2.58	0.03
Q61696	2.62	0.01
Q61838	2.64	0.00
Q62009	2.66	0.02
Q9D020	2.67	0.00
P32921	2.67	0.01
P50396	2.67	0.00
Q9CYZ2	2.67	0.04
P82349	2.69	0.02
Q80X19	2.69	0.03
Q9QZF2	2.72	0.00
P14602	2.72	0.00
P99024	2.81	0.04
Q9DBC7	2.81	0.00
Q6NTA4	2.82	0.02
Q9R0X4	2.83	0.03
Q9EQU5	2.85	0.03
Q99KC8	2.85	0.01
Q9D1H9	2.87	0.02
P97447	2.88	0.01
O70492	2.88	0.01
P62962	2.89	0.00
Q9DCN2	2.91	0.00
Q9QUM9	2.92	0.01
P62137	2.94	0.02
Q7TMM9	2.95	0.00
O55131	2.95	0.02
Q9QXT0	2.98	0.00
Q6P9Q4	2.98	0.01
Q9EQP2	2.98	0.00
P16110	3.02	0.03
Q91VI7	3.15	0.00
Q8BH61	3.15	0.01
P97429	3.17	0.01
P13745	3.21	0.02
Q8BW75	3.22	0.03

UniProt-AC	FC	P-Val
P24452	3.25	0.00
Q9R0P3	3.25	0.00
P63276	3.26	0.00
Q61656	3.26	0.01
Q5FW52	3.28	0.00
P10637	3.33	0.01
P28656	3.38	0.02
P59325	3.41	0.00
Q9DBS1	3.45	0.00
Q9JHR7	3.48	0.00
P42208	3.54	0.02
Q07076	3.56	0.00
P24270	3.64	0.02
O54950	3.71	0.00
Q9CQW2	3.77	0.00
Q921F2	3.87	0.05
Q9EQH3	3.91	0.00

UniProt-AC	FC	P-Val
Q64669	4.05	0.01
Q61147	4.11	0.02
Q5EBG6	4.13	0.01
P70168	5.35	0.01
Q60854	6.07	0.00
P23927	6.22	0.00
Q9D832	6.36	0.04
Q8VDC1	6.38	0.04
Q80XB4	6.88	0.00
Q8VEE1	7.53	0.00
P97425	7.66	0.00
Q9DCS2	7.67	0.04
Q9CQ22	8.34	0.00
Q9D6Y9	8.63	0.00
Q61878	8.96	0.00
Q99JI1	9.36	0.01
P35385	26.08	0.00

UniProt-AC	FC	P-Val
Q8BU85	0.57	0.02
Q3V1D3	0.64	0.00
Q8BGQ7	0.66	0.03
P63268	0.68	0.01
Q8C494	0.75	0.00
Q8VCR8	0.76	0.00
Q9DCL9	0.81	0.01
P56382	0.81	0.03
P11247	0.82	0.03
Q5XKE0	0.82	0.01
P58771	0.84	0.01
O88990	0.87	0.02
Q5SX39	0.88	0.00
Q91YE8	0.88	0.02
Q8R429	0.88	0.00
A2ASS6	0.91	0.01

Table S2: Significant upregulated Proteins overlapping with KEGG "pathway of neurodegeneration – multiple diseases"

UniProt Accession	Gene Name	fc	p-value	UniProt Accession	Gene Name	fc	p-value
Q9CQQ7	Atp5pb	1.52	0.0283	P63085	Mapk1	1.61	0.0317
P56480	Atp5f1b	1.50	0.0048	P47811	Mapk14	2.10	0.0164
Q9DCX2	Atp5pd	1.37	0.0070	P62192	Psmc1	1.38	0.0277
Q9DB20	Atp5po	1.41	0.0049	P62196	Psmc5	1.75	0.0272
Q03265	Atp5f1a	1.42	0.0011	P46471	Psmc2	1.52	0.0041
Q9D3D9	Atp5f1d	1.29	0.0123	P54775	Psmc4	1.88	0.0362
P03921	Mtnd5	1.65	0.0353	P62334	Psmc6	1.79	0.0097
Q91VD9	Ndufs1	1.34	0.0037	Q8BG32	Psmd11	1.42	0.0447
Q91WD5	Ndufs2	1.51	0.0229	Q9D8W5	Psmd12	1.68	0.0158
Q99LY9	Ndufs5	1.52	0.0139	Q9WVJ2	Psmd13	1.65	0.0055
P52503	Ndufs6	1.25	0.0299	Q8VDM4	Psmd2	1.87	0.0019
Q8K3J1	Ndufs8	1.39	0.0166	P14685	Psmd3	1.73	0.0006
Q91YT0	Ndufv1	1.49	0.0046	Q99JI4	Psmd6	1.87	0.0003
Q99LC3	Ndufa10	1.66	0.0074	O09061	Psmb1	1.39	0.0092
Q9D8B4	Ndufa11	1.47	0.0338	Q9R1P1	Psmb3	1.97	0.0170
Q7TMF3	Ndufa12	1.37	0.0116	P99026	Psmb4	1.85	0.0143
Q9ERS2	Ndufa13	1.44	0.0075	O55234	Psmb5	1.46	0.0026
Q9CQ75	Ndufa2	1.43	0.0141	P70195	Psmb7	1.50	0.0103
Q9CPP6	Ndufa5	1.70	0.0015	Q9R1P4	Psma1	1.51	0.0065
Q9CQZ5	Ndufa6	1.40	0.0044	O70435	Psma3	1.47	0.0014
Q9Z1P6	Ndufa7	1.47	0.0212	Q9R1P0	Psma4	2.14	0.0322
Q9DCJ5	Ndufa8	1.39	0.0008	Q9Z2U1	Psma5	1.45	0.0105
Q9DC69	Ndufa9	1.52	0.0019	Q9QUM9	Psma6	2.92	0.0061
Q9DCS9	Ndufb10	1.40	0.0054	Q9Z2U0	Psma7	1.39	0.0045
Q9CQC7	Ndufb4	1.31	0.0180	P63328	Ppp3ca	1.29	0.0386
Q9CQH3	Ndufb5	1.68	0.0122	P62983	Rps27a	1.66	0.0086
Q3UIU2	Ndufb6	1.66	0.0202	P48962	Slc25a4	1.38	0.0378
Q9CR61	Ndufb7	1.50	0.0339	P51881	Slc25a5	1.35	0.0034
Q9CQJ8	Ndufb9	1.57	0.0002	Q8K2B3	Sdha	1.52	0.0001
P63001	Rac1	1.30	0.0482	Q9CQA3	Sdhb	1.43	0.0019
Q921F2	Tardbp	3.87	0.0488	Q9CZB0	Sdhc	1.66	0.0212
Q9CQN1	Trap1	2.38	0.0031	Q9CXV1	Sdhd	1.36	0.0287
Q6PHZ2	Camk2d	2.20	0.0003	P68369	Tuba1a	1.79	0.0023
O35350	Capn1	1.42	0.0049	P68368	Tuba4a	1.79	0.0000
O08529	Capn2	2.10	0.0074	Q9JJZ2	Tuba8	2.35	0.0007
P24270	Cat	3.64	0.0212	Q7TMM9	Tubb2a	2.95	0.0016
P00158	Mt-Cyb	1.79	0.0220	P99024	Tubb5	2.81	0.0436
P00405	Mtco2	1.47	0.0011	Q922F4	Tubb6	1.80	0.0376
P19783	Cox4i1	1.38	0.0231	Q9DB77	Uqcrc2	1.43	0.0009
Q9CPQ1	Cox6c	1.31	0.0352	Q9D855	Uqcrb	1.39	0.0059
P56391	Cox6b1	1.44	0.0036	Q9CZ13	Uqcrc1	1.45	0.0049
P62897	Cycs	1.42	0.0057	Q9CR68	Uqcrfs1	1.34	0.0226
Q9D0M3	Cyc1	1.41	0.0013	Q9CQ69	Uqcrq	1.24	0.0389
Q6ZWX6	Eif2s1	1.47	0.0055	Q8R1I1	Uqcr10	1.43	0.0465
P11352	Gpx1	2.45	0.0279	P68037	Ube2l3	1.25	0.0091
P20029	Hspa5	1.44	0.0027	Q01853	Vcp	1.51	0.0043
O08756	Hsd17b10	1.60	0.0261	Q60930	Vdac2	1.50	0.0007
P10637	Mapt	3.33	0.0052	Q60931	Vdac3	1.28	0.0412
